# Supplementary figures and images for: Massive analysis of 64,628 bacterial genomes to decipher water reservoir and origin of mobile colistin resistance genes: is there another role for these enzymes?
Source: Sci Rep. 2020 Apr 6;10:5970. doi: 10.1038/s41598-020-63167-5 (PMC7136264; doi:10.1038/s41598-020-63167-5)

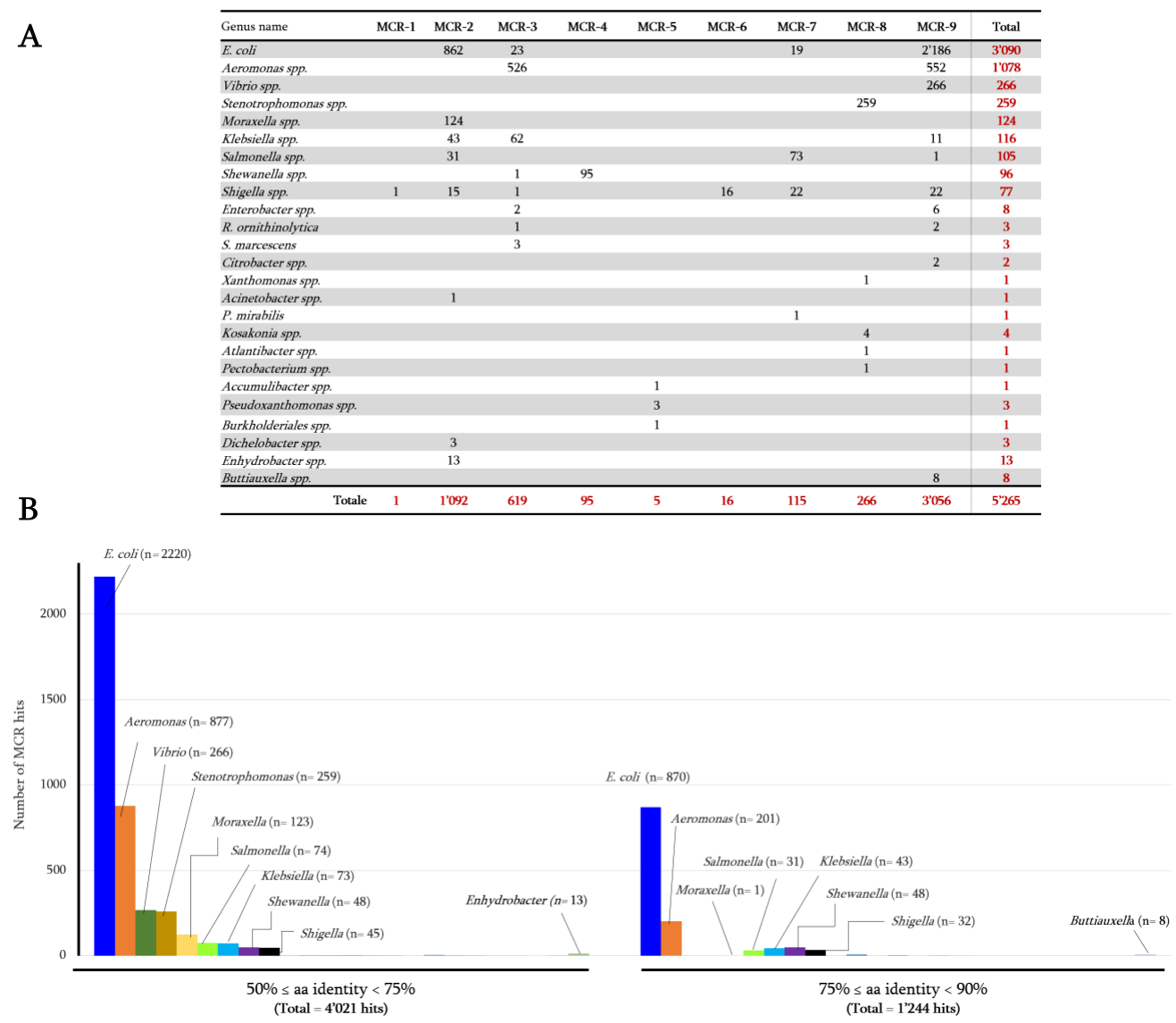

Supplement: Supplementary file 1 — Supplementary Information. [file 41598_2020_63167_MOESM1_ESM.tif]

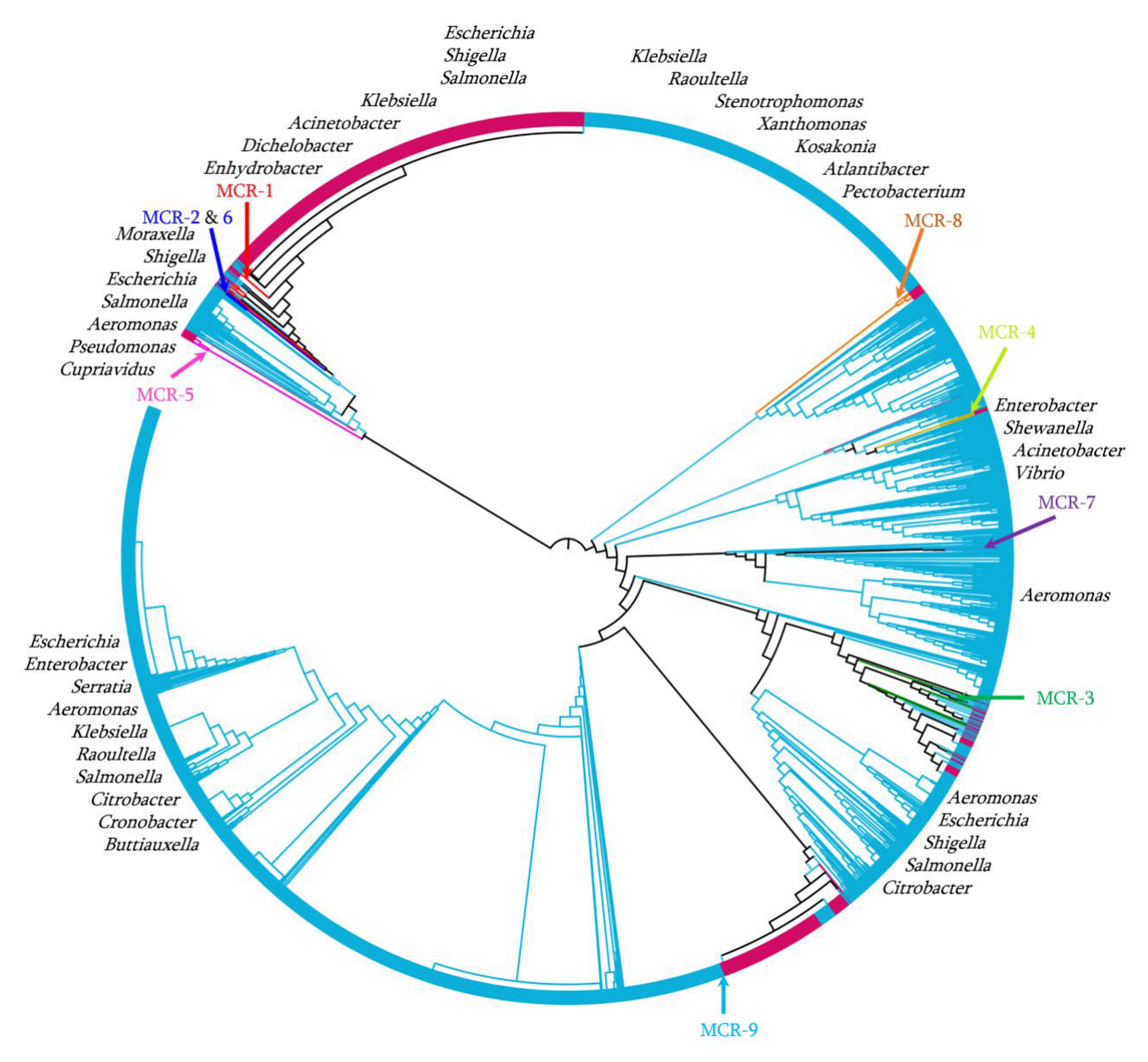

Supplement: Supplementary file 2 — Supplementary Information 2. [file 41598_2020_63167_MOESM2_ESM.tif]

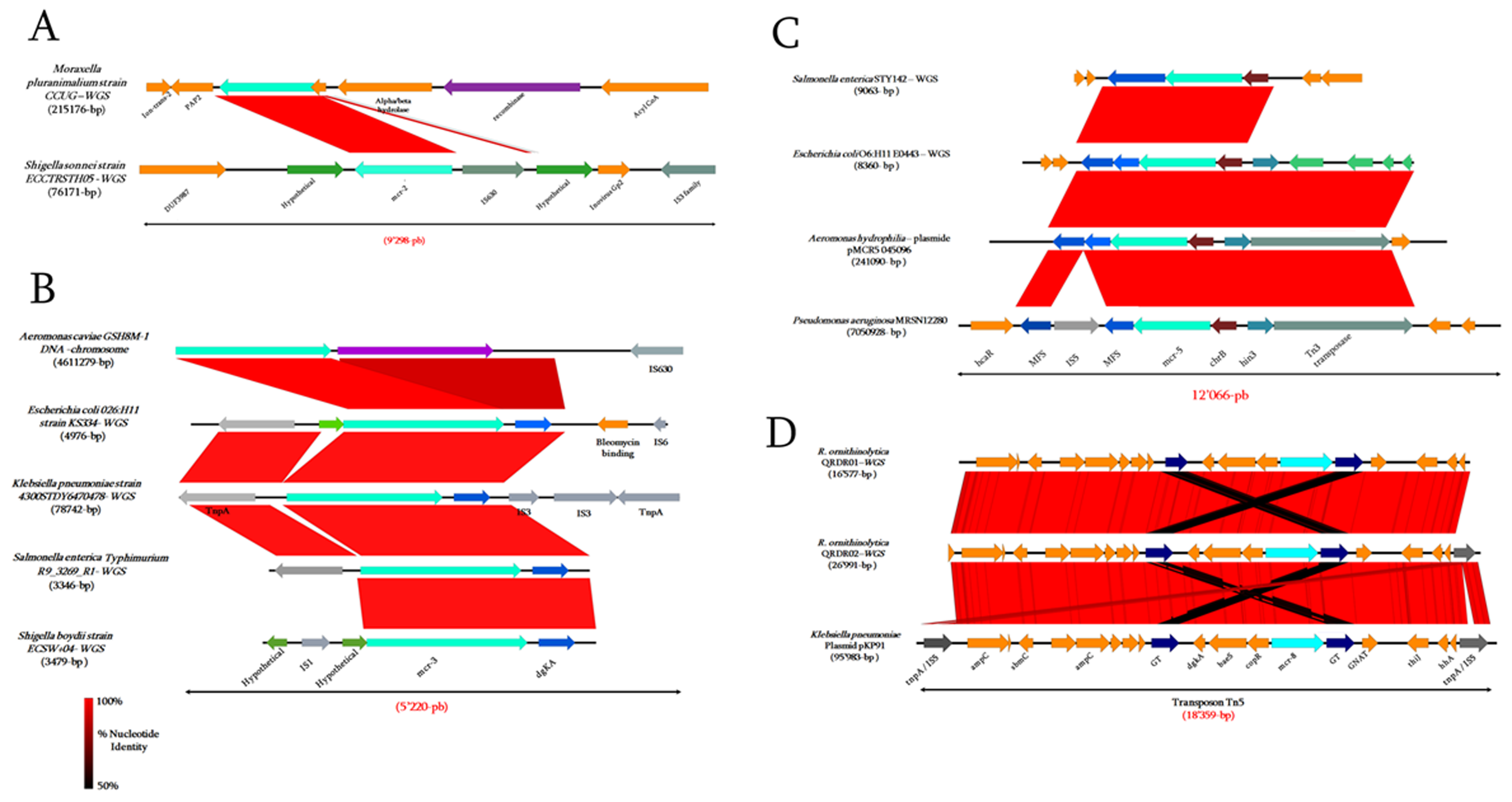

Supplement: Supplementary file 3 — Supplementary Information 3. [file 41598_2020_63167_MOESM3_ESM.tif]

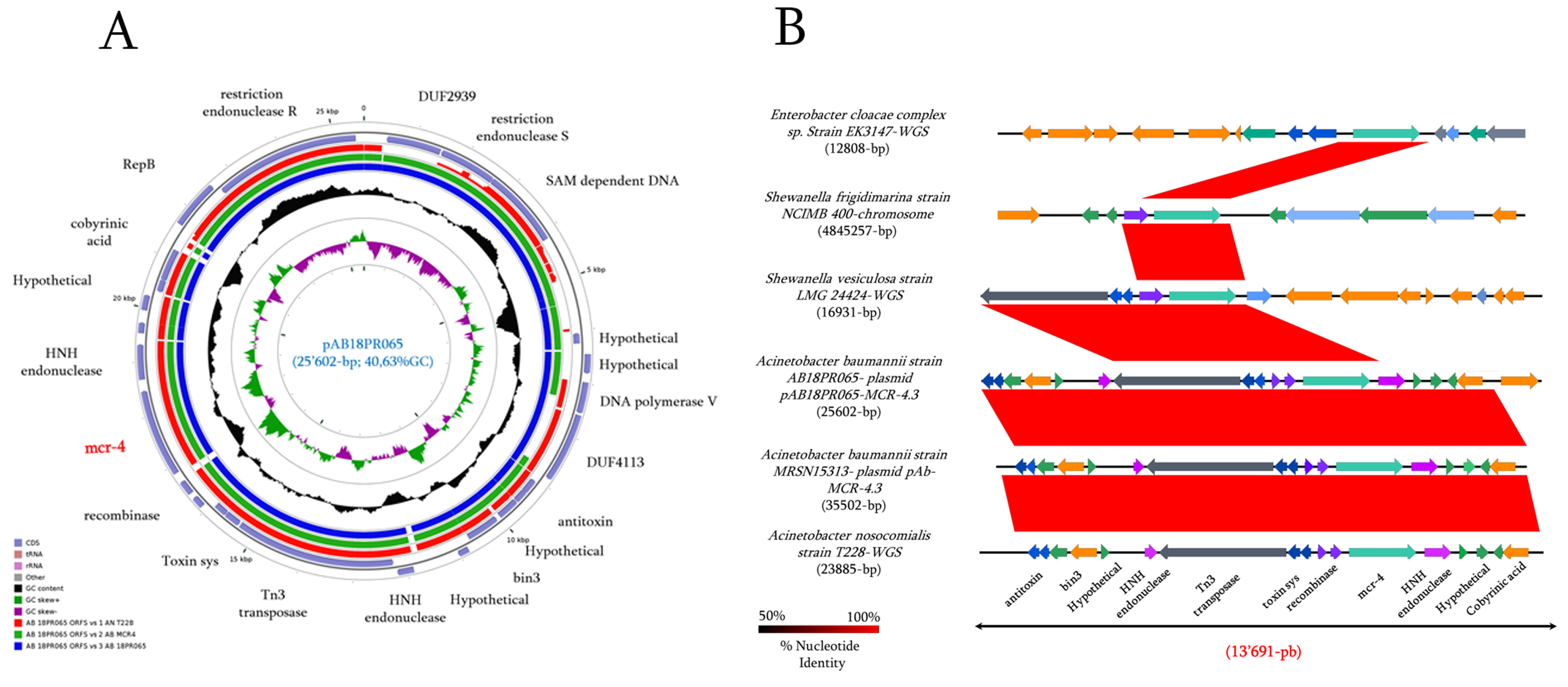

Supplement: Supplementary file 5 — Supplementary Information 5. [file 41598_2020_63167_MOESM5_ESM.tif]
