# Supplementary material for: Massive analysis of 64,628 bacterial genomes to decipher water reservoir and origin of mobile colistin resistance genes: is there another role for these enzymes?
Source: Sci Rep. 2020 Apr 6;10:5970. doi: 10.1038/s41598-020-63167-5 (PMC7136264; doi:10.1038/s41598-020-63167-5)
Supplement: Supplementary file 8 — Supplementary Information 8. [file 41598_2020_63167_MOESM8_ESM.doc]

**Suppl. Table S3:** General features of depicted genes on the different genetic environment figures.

|  | **Gene name** | **Length (bp)** | **%GC content** | **Function** |
| --- | --- | --- | --- | --- |
| MCR-2 | DUF3987 | 1434 | 42.82 | DUF3987 domain-containing protein |
| HP | 648 | 39.04 | hypothetical protein |
| MCR-2 | 1617 | 47.19 | putative phosphatidylethanolamine transferase Mcr-1 |
| IS630 | 1032 | 51.36 | IS630-like element ISSpu2 family transposase |
| HP | 648 | 39.04 | hypothetical protein |
| inovirus Gp2 | 534 | 45.32 | inovirus Gp2 family protein |
| IS3 | 906 | 54.86 | IS3 family transposase |
| Ion_trans2 | 408 | 39.95 | two pore domain potassium channel family protein |
| PAP2 | 705 | 46.10 | PAP2 family lipid A phosphatase |
| mcr1 | 1617 | 46.94 | putative phosphatidylethanolamine transferase Mcr-1 |
| alpha/beta hydrolase | 1563 | 48.62 | alpha/beta hydrolase |
| recombinase | 2268 | 46.34 | Recombinase |
| acyl-CoA | 1776 | 50.17 | acyl-CoA dehydrogenase |
| MCR-3 | TnpA | 651 | 55.30 | TnpA transposase |
| IS3 | 663 | 58.22 | IS3 family transposase |
| Transposase | 315 | 56.19 | Transposase |
| MCR-3 | 1626 | 41.76 | Phosphoethanolamine transferase EptA |
| TnpA | 795 | 60.63 | TnpA transposase |
| dgkA | 381 | 45.14 | Diacylglycerol kinase |
| HP | 261 | 40.61 | hypothetical protein |
| IS1 | 294 | 52.38 | IS1 family transposase |
| HP | 216 | 54.63 | hypothetical protein |
| IS6 | 132 | 48.48 | IS6 family transposase |
| bleomycin binding | 318 | 41.19 | Bleomycin resistance protein |
| HP | 261 | 40.61 | hypothetical protein |
| MCR-4 | antitoxin | 288 | 38.19 | type II toxin-antitoxin system Phd/YefM family antitoxin |
| antitoxin | 249 | 40.56 | type II toxin-antitoxin system Phd/YefM family antitoxin |
| Hypothetical | 432 | 41.90 | hypothetical protein |
| bin3 | 654 | 44.04 | Putative transposon Tn552 DNA-invertase bin3 |
| Hypothetical | 210 | 38.10 | hypothetical protein |
| HNH endonuclease | 309 | 33.01 | HNH endonuclease |
| Tn3 transposase | 3087 | 41.43 | Tn3 transposase |
| antitoxin | 312 | 43.27 | type II toxin-antitoxin system Phd/YefM family antitoxin |
| antitoxin | 261 | 40.23 | type II toxin-antitoxin system Phd/YefM family antitoxin |
| recombinase | 222 | 45.95 | recombinase |
| recombinase | 279 | 41.94 | recombinase |
| MCR-4 | 1626 | 40.10 | Phosphoethanolamine transferase EptA |
| HNH endonuclease | 651 | 38.40 | HNH endonuclease |
| Hypothetical | 204 | 37.75 | hypothetical protein |
| Hypothetical | 291 | 37.46 | hypothetical protein |
| Hypothetical | 237 | 37.97 | hypothetical protein |
| Cobyrinic acid | 630 | 40.00 | Cobyrinic acid |
| MCR-5 | MFS | 657 | 64.99 | [MFS transporter](https://blast.ncbi.nlm.nih.gov/Blast.cgi#alnHdr_AVN57281) |
| IS5 | 963 | 67.81 | IS5 family transposase |
| MFS | 624 | 64.74 | [MFS transporter](https://blast.ncbi.nlm.nih.gov/Blast.cgi#alnHdr_AVN57281) |
| MCR-5 | 1644 | 55.47 | Phosphoethanolamine transferase EptA |
| chrB | 543 | 56.72 | Protein ChrB |
| hin | 561 | 61.85 | DNA-invertase hin |
| Tn3 transposase | 2967 | 63.67 | Tn3 transposase |
| MCR-8 | tnpA / IS5 | 924 | 52.71 | IS5 transposase family |
| hhA | 204 | 38.73 | hemolysin expression modulator Hha |
| thiJ | 699 | 47.78 | thiamine biosynthesis protein ThiJ |
| GNAT | 510 | 53.14 | Acetyltransferase (GNAT) family protein |
| GT | 912 | 37.06 | Glycosyl transferase |
| mcr-8 | 1698 | 40.40 | phosphoethanolamine--lipid A transferase MCR-8.1 |
| copR | 696 | 47.84 | Transcriptional activator protein CopR |
| baeS | 1230 | 43.01 | Integral membrane sensor signal transduction histidine kinase |
| dgkA | 390 | 39.23 | Diacylglycerol kinase |
| GT | 900 | 41.89 | Glycosyl transferase |
| - | 210 | 45.24 | Transcriptional regulator |
| - | 534 | 48.50 | hypothetical protein |
| - | 357 | 42.58 | hypothetical protein |
| ampC | 1092 | 54.85 | Beta-lactamase precursor |
| - | 807 | 52.66 | MltA-interacting protein MipA |
| sbmC | 471 | 46.28 | DNA gyrase inhibitor |
| - | 90 | 40.00 | hypothetical protein |
| ampC | 1365 | 48.50 | Beta-lactamase precursor |
| tnpA / IS5 | 924 | 53.57 | IS5 transposase family |
| MCR-9 | *ATP/GTP* | 1206 | 47.51 | ATP/GTP-binding protein |
| *DUF* | 819 | 49.21 | DUF4942 domain-containing protein |
| *HP* | 168 | 42.26 | Hypothetical protein |
| *rcnR* | 273 | 43.96 | Ni(II)/Co(II)-binding transcriptional repressor RcnR |
| *rcnA* | 1116 | 49.73 | Nickel/cobalt efflux protein RcnA |
| *pcoE* | 435 | 47.59 | Putative copper-binding protein PcoE |
| *cusS* | 1347 | 47.14 | Sensor kinase CusS |
| *IS5* | 924 | 54.11 | IS5 family transposase |
| *mcr-9* | 1620 | 44.88 | Phosphoethanolamine transferase EptA |
| *wbuC* | 477 | 53.04 | Cupin fold metalloprotein, WbuC family |
| *hAMP* | 1350 | 50.37 | HAMP domain-containing histidine kinase |
| *Res* | 669 | 53.21 | Response regulator |
| *ATPAse* | 969 | 53.77 | AAA family ATPase |
| *IS481* | 885 | 55.03 | IS481 family transposase |
| *IS6* | 705 | 53.33 | IS6 family transposase |
| *IS110* | 1023 | 49.76 | IS110 family transposase |
| *toxin-antitoxin* | 330 | 50.00 | Type II toxin-antitoxin system RelE/ParE family toxin |
| *ardK* | 342 | 43.57 | Transcriptional regulator ArdK |
| *zinc M* | 792 | 52.02 | Zinc metalloprotease |
| *aph(6)-I* | 837 | 55.91 | APH(6)-I family aminoglycoside O-phosphotransferase |
| *aph(3'')-Ib* | 804 | 56.22 | Aminoglycoside O-phosphotransferase APH(3'')-Ib |
| *xerD* | 1014 | 61.14 | Tyrosine recombinase XerD |
| *endonuclease* | 642 | 49.07 | Restriction endonuclease |
