# Supplementary material for: Massive analysis of 64,628 bacterial genomes to decipher water reservoir and origin of mobile colistin resistance genes: is there another role for these enzymes?
Source: Sci Rep. 2020 Apr 6;10:5970. doi: 10.1038/s41598-020-63167-5 (PMC7136264; doi:10.1038/s41598-020-63167-5)
Supplement: Supplementary file 9 — Supplementary Information 9. [file 41598_2020_63167_MOESM9_ESM.doc]

**Massive analysis of 64’628 bacterial genomes to decipher water reservoir and origin of mobile colistin resistance genes: is there another role for these enzymes?**

Mariem Ben Khedher^1^, Sophie Alexandra Baron^1^, Toilhata Riziki^1^, Raymond Ruimy ^3^, Didier Raoult^1,2^, Seydina M. Diene^1,2^*, Jean-Marc Rolain^1,2^*

*** Corresponding authors:**

**Address :** IHU-Mediterranee Infection, 19-21 Bd Jean Moulin, 13005 Marseille, France

**E-mail-1**: [jean-marc.rolain@univ-amu.fr](mailto:jean-marc.rolain@univ-amu.fr)

**E-mail-2** : [seydina.diene@univ-amu.fr](mailto:seydina.diene@univ-amu.fr)


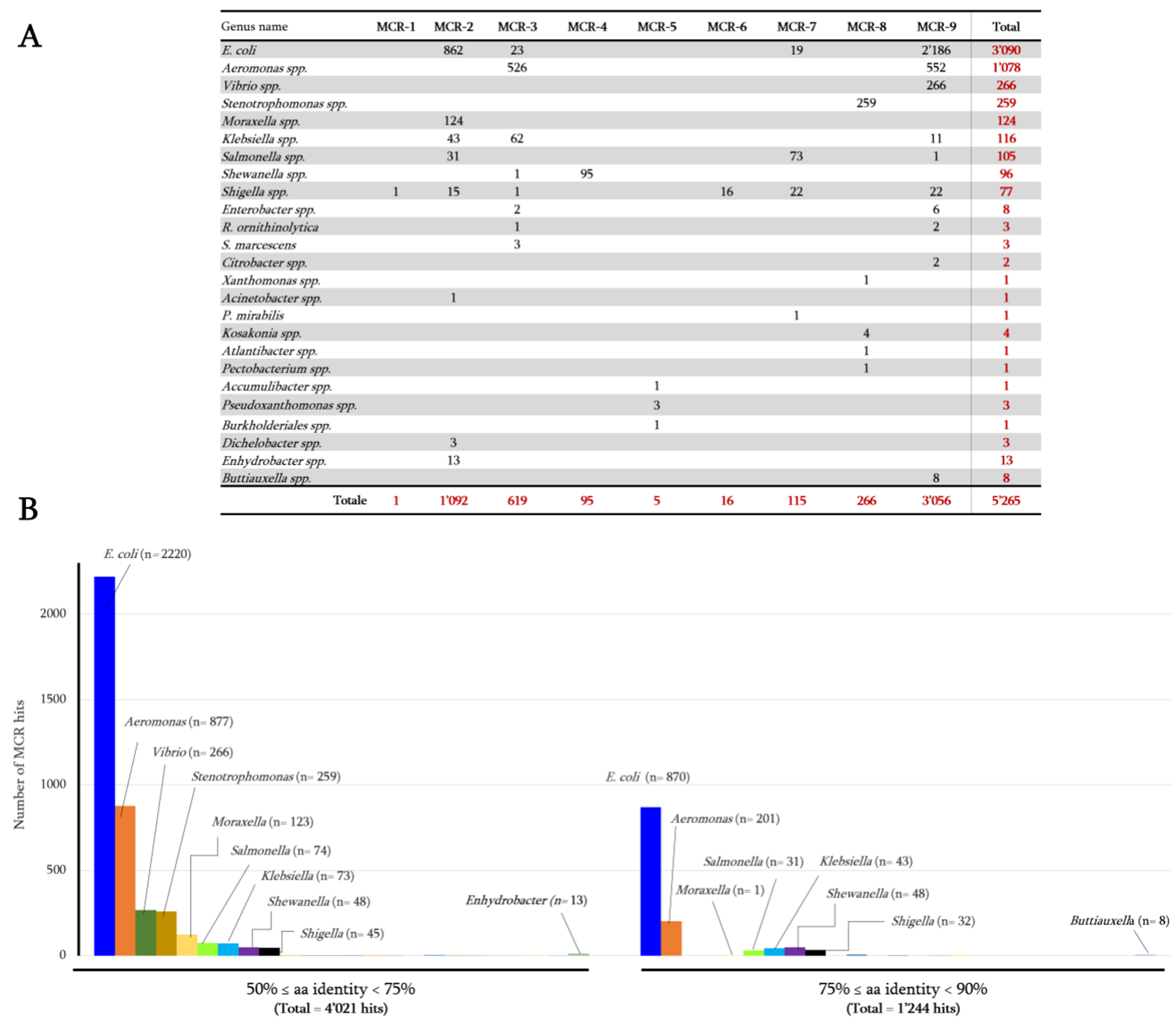


**Supplementary Fig. 1**: Distribution of MCR hits with aa identity between 50% to 90% with all MCR reference variants. (A) Table showing the distribution of the total 5’230 hits among the 16 out of the 32 bacterial genera analyzed. (B) Graph showing the distribution of these sequences according to bacterial genera and to aa identity between 50 and 75% and those with identity from 75 to 90%.

**
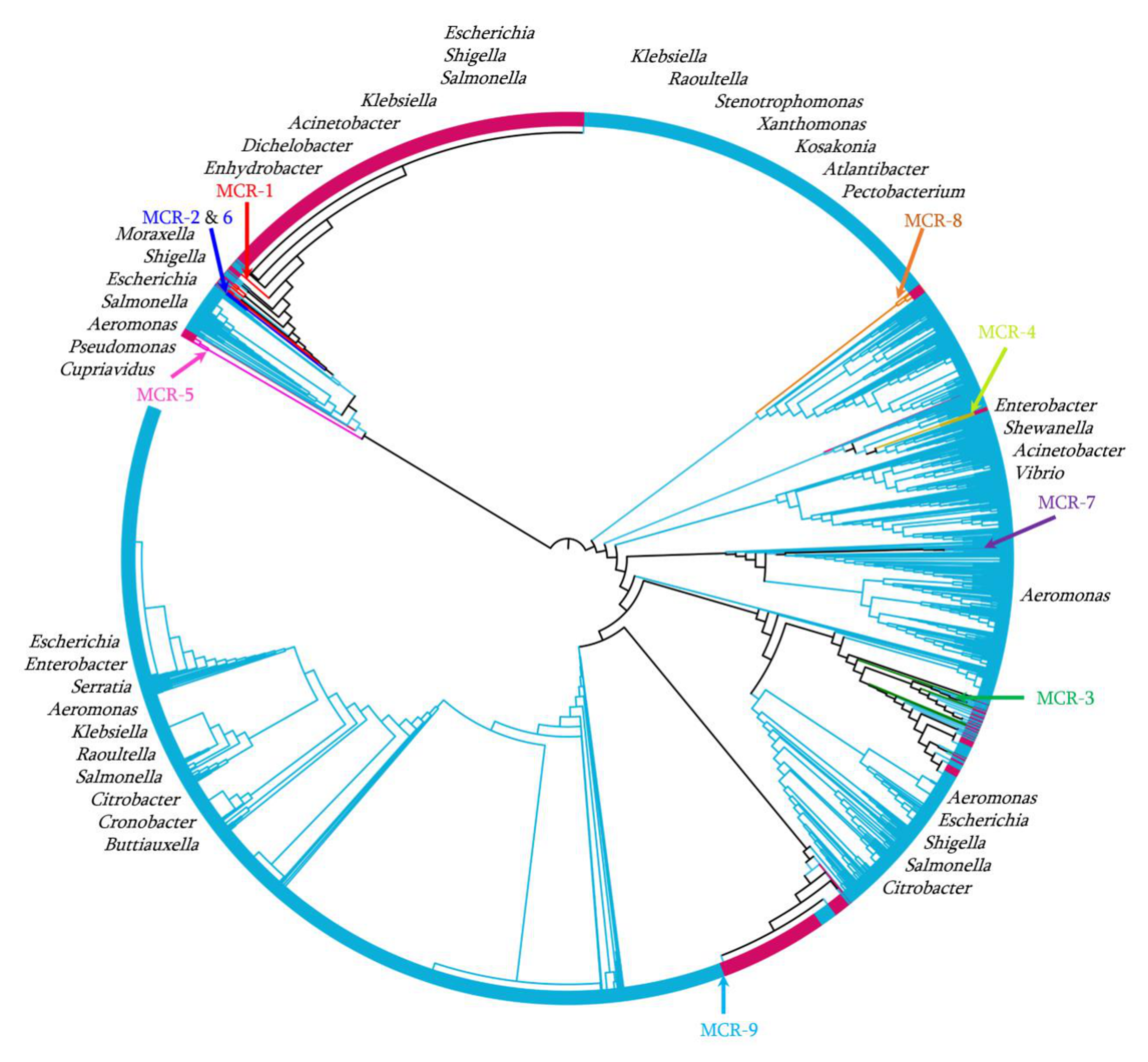
**

**Supplementary Fig. 2**: Phylogenetic tree performed from MCR variant hits (n=6’648 proteins) with aa identity between 50% and 100% and alignment ≥ 90%.


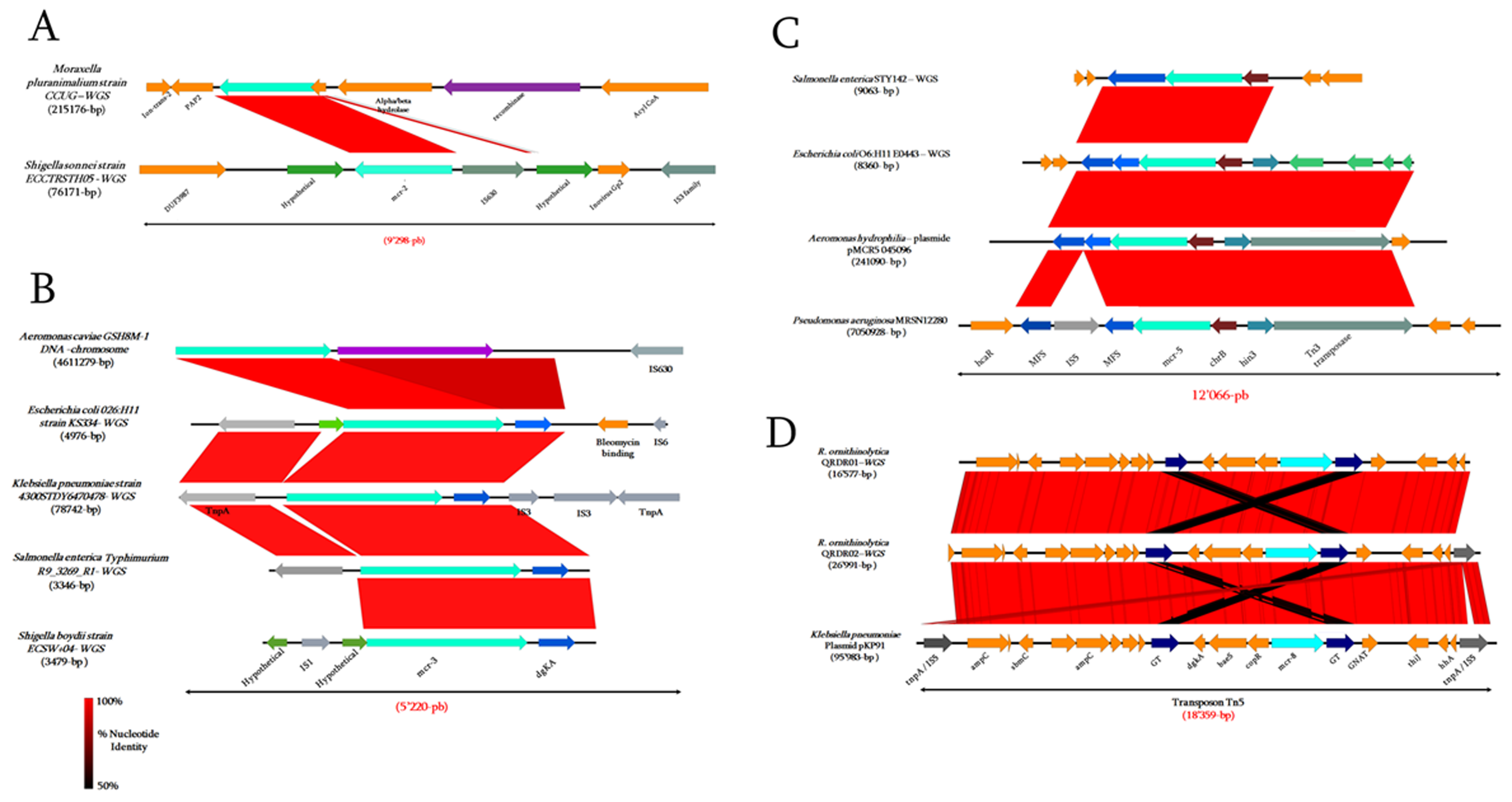


**Supplementary Fig. 3:** Genetic environment analysis of *mcr-2*; *mcr-3*; *mcr-5* and *mcr-8* in bacterial genomes. **(A)** Comparison of the *mcr-2* identified in *M. pluranimalium* and *S. sonnei*. **(B)** Genetic environment of *mcr-5* identified in. *S. enterica*; *E. coli*; *A. hydrophilia* and *P. aeruginosa*. **(C)** Genetic environment of *mcr-3* identified in *A. caviae. E. coli. K. pneumoniae. S. enterica Typhimurium* and *S. boydii*. **(D)** Comparison of the *mcr*-8 identified in *K. pneumoniae* and *R. ornithinolytica.* The arrows indicate the positions and directions of the ORFs. Regions of more than 90% homology are marked by red shading.

**

**

**Supplementary Fig. 4:** Genetic environment of *mcr-9* gene in different bacterial genomes of different bacterial species. Gene size, % GC content and predicted function are indicated in **Supplementary Table 3.**

**
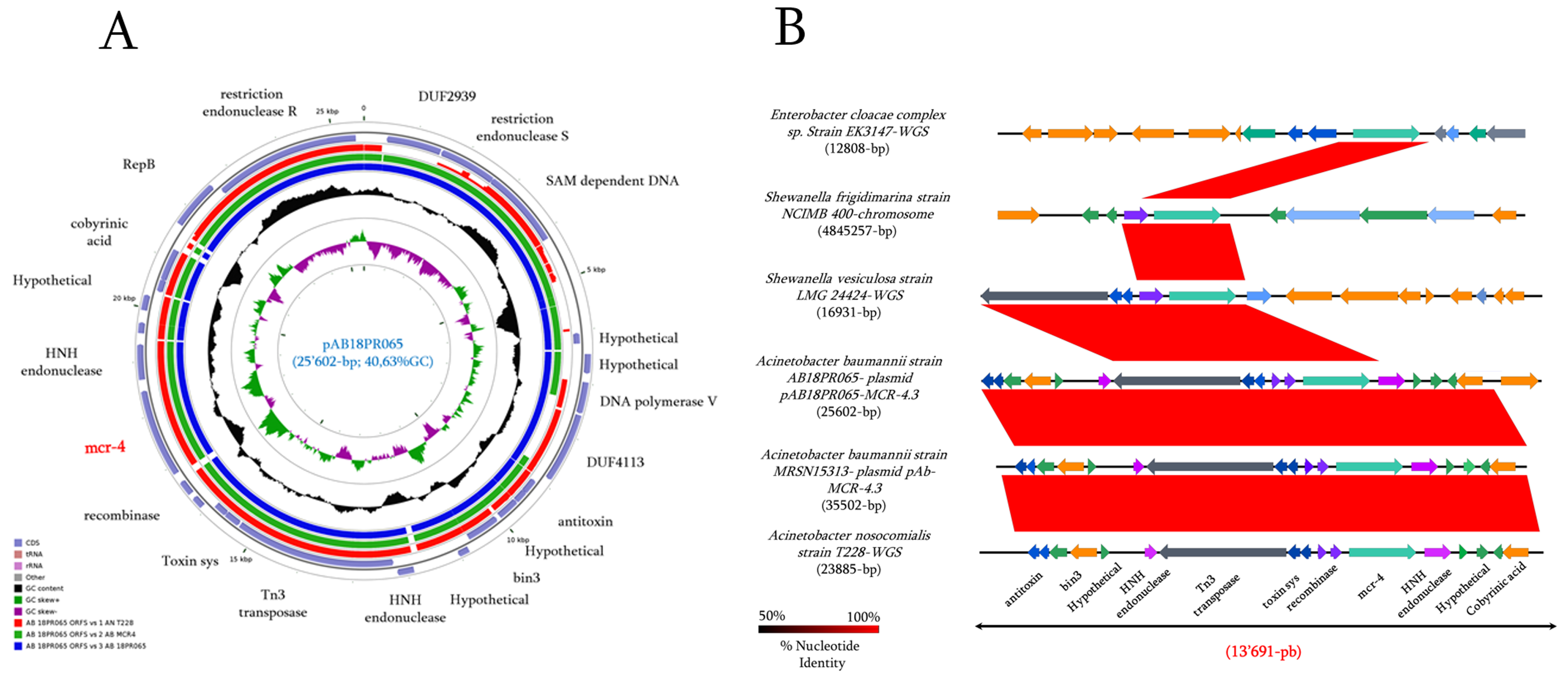
**

**Supplementary Fig. 5:** Comparison of the genetic environment of *mcr*-4 gene in bacterial genomes. **(A)** Comparison of the three plasmids harboring *mcr*-4 gene identified in *A. baumannii* and *A. nosocomialis* using CGView software. **(B)** Genetic environment of the *mcr*-4 gene. Gene size, % GC content and predicted function are indicated in **Supplementary Table 3.**

**Supplementary Table 1:** MCR variants hits of available bacterial genomes from the different subtrees presented in Figure 2.

| **Genus name** | **Source** | **MCR variants** | **Species** | **Complete Genomes** | **WGS** | **GC%** | **MCR-1** | **MCR-2** | **MCR-3** | **MCR-4** | **MCR-5** | **MCR-6** | **MCR-7** | **MCR-8** | **MCR-9** | **Total** |
| --- | --- | --- | --- | --- | --- | --- | --- | --- | --- | --- | --- | --- | --- | --- | --- | --- |
| ***Enhydrobacter*** | Sea water | MCR-1, 2 & 6 | 2 | - | 15 | 43.6 | - | 12 | - | - | - | - | - | - | - | **12** |
| ***Dichelobacter*** | Sea water | MCR-1, 2 & 6 | 1 | 1 | 2 | 44.4 | - | 3 | - | - | - | - | - | - | - | **3** |
| ***Methylophilaceae*** | Freshwater | MCR-1, 2 & 6 | 24 | 6 | 62 | 50.3 | - | 57 | - | - | - | - | - | - | - | **57** |
| ***Limnobacter*** | Sea water, environment, soil | MCR-1, 2 & 6 | 2 | - | 13 | 52.2 | - | 12 | - | - | - | - | - | - | - | **12** |
| ***Buttiauxella*** | Soil, animal, human | MCR-3 & 7 & 9 | 8 | 1 | 9 | 52.6 | - | - | 1 | - | - | - | - | - | 13 | **14** |
| ***Salinicola*** | Sea water | MCR-5 | 7 | 1 | 17 | 63.6 | - | - | - | - | 3 | - | - | - | - | **3** |
| ***Idiomarina*** | Sea water | MCR-5 | 28 | 5 | 45 | 47 | - | - | - | - | - | - | - | - | 2 | **2** |
| ***Halomonas*** | Sea water | MCR-5 | 59 | 18 | 121 | 55.9 | - | - | - | - | 46 | - | - | - | - | **46** |
| ***Burkholderiales*** | Soil, water, human | MCR-5 | 2 | 124 | 1’383 | 66.4 | - | - | - | - | 2 | - | - | - | 1 | **3** |
| ***Luteimonas*** | Sea water, environment | MCR-5 | 4 | 4 | 7 | 69.3 | - | - | - | - | 5 | - | - | - | - | **5** |
| ***Lysobacter*** | Soil | MCR-5 | 15 | 10 | 34 | 68.3 | - | - | - | - | 34 | - | - | - | - | **34** |
| ***Arenimonas*** | Sea water, environment, soil | MCR-5 | 7 | - | 8 | 70 | - | - | - | - | 5 | - | - | - | - | **5** |
| ***Pseudoxanthomonas*** | Soil, plant | MCR-5 | 7 | 3 | 33 | 69 | - | - | - | - | 42 | - | - | - | - | **42** |
| ***Caldimonas*** | Hot spring (water) | MCR-5 | 2 | - | 2 | 66 | - | - | - | - | 2 | - | - | - | - | **2** |
| ***Rubrivivax*** | Hot spring (water) | MCR-5 | 3 | 1 | 13 | 68.4 | - | - | - | - | 11 | - | - | - | 1 | **12** |
| ***Sphaerotilus*** | Rivers, sewage | MCR-5 | 2 | - | 3 | 69.9 | - | - | - | - | 2 | - | - | - | - | **2** |
| ***Accumulibacter*** | Water | MCR-5 | 3 | 1 | 24 | 62.1 | - | - | - | - | 2 | - | - | - | - | **2** |
| ***Leptothrix*** | Groundwater | MCR-5 | 3 | 1 | 2 | 68.9 | - | - | - | - | 2 | - | - | - | - | **2** |
| ***Hylemonella*** | Wastewater | MCR-5 | 2 | - | 6 | 55.2 | - | - | - | - | 2 | - | - | - | - | **2** |
| ***Herminiimonas*** | Bottled mineral water | MCR-5 | 4 | 2 | 3 | 56.4 | - | - | - | - | 4 | - | - | - | - | **4** |
| ***Dechloromonas*** | Environment, human gut | MCR-5 | 4 | 2 | 9 | 61 | - | - | - | - | 2 | - | - | - | - | **2** |
| ***Rhodoferax*** | Seawater | MCR-5 | 7 | 6 | 6 | 61.4 | - | - | - | - | 4 | - | - | - | - | **4** |
| ***Acidovorax*** | Soil | MCR-5 | 18 | 14 | 73 | 64.8 | - | - | - | - | 25 | - | - | - | 7 | **32** |
| ***Thauera*** | Hot spring (water) | MCR-5 | 13 | 6 | 14 | 66.4 | - | - | - | - | 12 | - | - | - | - | **12** |
| ***Pectobacterium*** | Soil, plant | MCR-8 | 12 | 26 | 110 | 51.8 | - | - | - | - | - | - | - | 48 | 88 | **136** |
| ***Atlantibacter*** | Human, soil | MCR-8 | 2 | 1 | 5 | 54.1 | - | - | - | - | - | - | - | 3 | 1 | **4** |
| ***Kosakonia*** | Environment, soil | MCR-8 | 9 | 8 | 17 | 53.9 | - | - | - | - | - | - | - | 30 | 1 | **31** |
| **Total** |  |  | **250** | **241** | **2’036** | **-** | **0** | **84** | **1** | **0** | **205** | **0** | **0** | **81** | **114** | **485** |

**Supplementary Table 2:** Detailed metadata of all MCR-producer strains including strain names, aa identity with MCR variants, Bioproject number, collection dates, geographical location, isolation sources, and host organisms.

**>>> Cf. Excel file**

**Supplementary Table 3:** General features of depicted genes on the different genetic environment figures.

|  | **Gene name** | **Length (bp)** | **%GC content** | **Function** |
| --- | --- | --- | --- | --- |
| MCR-2 | DUF3987 | 1434 | 42.82 | DUF3987 domain-containing protein |
|  | HP | 648 | 39.04 | hypothetical protein |
|  | MCR-2 | 1617 | 47.19 | putative phosphatidylethanolamine transferase Mcr-1 |
|  | IS630 | 1032 | 51.36 | IS630-like element ISSpu2 family transposase |
|  | HP | 648 | 39.04 | hypothetical protein |
|  | inovirus Gp2 | 534 | 45.32 | inovirus Gp2 family protein |
|  | IS3 | 906 | 54.86 | IS3 family transposase |
|  | Ion_trans2 | 408 | 39.95 | two pore domain potassium channel family protein |
|  | PAP2 | 705 | 46.10 | PAP2 family lipid A phosphatase |
|  | mcr1 | 1617 | 46.94 | putative phosphatidylethanolamine transferase Mcr-1 |
|  | alpha/beta hydrolase | 1563 | 48.62 | alpha/beta hydrolase |
|  | recombinase | 2268 | 46.34 | Recombinase |
|  | acyl-CoA | 1776 | 50.17 | acyl-CoA dehydrogenase |
| MCR-3 | TnpA | 651 | 55.30 | TnpA transposase |
|  | IS3 | 663 | 58.22 | IS3 family transposase |
|  | Transposase | 315 | 56.19 | Transposase |
|  | MCR-3 | 1626 | 41.76 | Phosphoethanolamine transferase EptA |
|  | TnpA | 795 | 60.63 | TnpA transposase |
|  | dgkA | 381 | 45.14 | Diacylglycerol kinase |
|  | HP | 261 | 40.61 | hypothetical protein |
|  | IS1 | 294 | 52.38 | IS1 family transposase |
|  | HP | 216 | 54.63 | hypothetical protein |
|  | IS6 | 132 | 48.48 | IS6 family transposase |
|  | bleomycin binding | 318 | 41.19 | Bleomycin resistance protein |
|  | HP | 261 | 40.61 | hypothetical protein |
| MCR-4 | antitoxin | 288 | 38.19 | type II toxin-antitoxin system Phd/YefM family antitoxin |
|  | antitoxin | 249 | 40.56 | type II toxin-antitoxin system Phd/YefM family antitoxin |
|  | Hypothetical | 432 | 41.90 | hypothetical protein |
|  | bin3 | 654 | 44.04 | Putative transposon Tn552 DNA-invertase bin3 |
|  | Hypothetical | 210 | 38.10 | hypothetical protein |
|  | HNH endonuclease | 309 | 33.01 | HNH endonuclease |
|  | Tn3 transposase | 3087 | 41.43 | Tn3 transposase |
|  | antitoxin | 312 | 43.27 | type II toxin-antitoxin system Phd/YefM family antitoxin |
|  | antitoxin | 261 | 40.23 | type II toxin-antitoxin system Phd/YefM family antitoxin |
|  | recombinase | 222 | 45.95 | recombinase |
|  | recombinase | 279 | 41.94 | recombinase |
|  | MCR-4 | 1626 | 40.10 | Phosphoethanolamine transferase EptA |
|  | HNH endonuclease | 651 | 38.40 | HNH endonuclease |
|  | Hypothetical | 204 | 37.75 | hypothetical protein |
|  | Hypothetical | 291 | 37.46 | hypothetical protein |
|  | Hypothetical | 237 | 37.97 | hypothetical protein |
|  | Cobyrinic acid | 630 | 40.00 | Cobyrinic acid |
| MCR-5 | MFS | 657 | 64.99 | MFS transporter |
|  | IS5 | 963 | 67.81 | IS5 family transposase |
|  | MFS | 624 | 64.74 | MFS transporter |
|  | MCR-5 | 1644 | 55.47 | Phosphoethanolamine transferase EptA |
|  | chrB | 543 | 56.72 | Protein ChrB |
|  | hin | 561 | 61.85 | DNA-invertase hin |
|  | Tn3 transposase | 2967 | 63.67 | Tn3 transposase |
| MCR-8 | tnpA / IS5 | 924 | 52.71 | IS5 transposase family |
|  | hhA | 204 | 38.73 | hemolysin expression modulator Hha |
|  | thiJ | 699 | 47.78 | thiamine biosynthesis protein ThiJ |
|  | GNAT | 510 | 53.14 | Acetyltransferase (GNAT) family protein |
|  | GT | 912 | 37.06 | Glycosyl transferase |
|  | mcr-8 | 1698 | 40.40 | phosphoethanolamine--lipid A transferase MCR-8.1 |
|  | copR | 696 | 47.84 | Transcriptional activator protein CopR |
|  | baeS | 1230 | 43.01 | Integral membrane sensor signal transduction histidine kinase |
|  | dgkA | 390 | 39.23 | Diacylglycerol kinase |
|  | GT | 900 | 41.89 | Glycosyl transferase |
|  | - | 210 | 45.24 | Transcriptional regulator |
|  | - | 534 | 48.50 | hypothetical protein |
|  | - | 357 | 42.58 | hypothetical protein |
|  | ampC | 1092 | 54.85 | Beta-lactamase precursor |
|  | - | 807 | 52.66 | MltA-interacting protein MipA |
|  | sbmC | 471 | 46.28 | DNA gyrase inhibitor |
|  | - | 90 | 40.00 | hypothetical protein |
|  | ampC | 1365 | 48.50 | Beta-lactamase precursor |
|  | tnpA / IS5 | 924 | 53.57 | IS5 transposase family |
| MCR-9 | *ATP/GTP* | 1206 | 47.51 | ATP/GTP-binding protein |
|  | *DUF* | 819 | 49.21 | DUF4942 domain-containing protein |
|  | *HP* | 168 | 42.26 | Hypothetical protein |
|  | *rcnR* | 273 | 43.96 | Ni(II)/Co(II)-binding transcriptional repressor RcnR |
|  | *rcnA* | 1116 | 49.73 | Nickel/cobalt efflux protein RcnA |
|  | *pcoE* | 435 | 47.59 | Putative copper-binding protein PcoE |
|  | *cusS* | 1347 | 47.14 | Sensor kinase CusS |
|  | *IS5* | 924 | 54.11 | IS5 family transposase |
|  | *mcr-9* | 1620 | 44.88 | Phosphoethanolamine transferase EptA |
|  | *wbuC* | 477 | 53.04 | Cupin fold metalloprotein, WbuC family |
|  | *hAMP* | 1350 | 50.37 | HAMP domain-containing histidine kinase |
|  | *Res* | 669 | 53.21 | Response regulator |
|  | *ATPAse* | 969 | 53.77 | AAA family ATPase |
|  | *IS481* | 885 | 55.03 | IS481 family transposase |
|  | *IS6* | 705 | 53.33 | IS6 family transposase |
|  | *IS110* | 1023 | 49.76 | IS110 family transposase |
|  | *toxin-antitoxin* | 330 | 50.00 | Type II toxin-antitoxin system RelE/ParE family toxin |
|  | *ardK* | 342 | 43.57 | Transcriptional regulator ArdK |
|  | *zinc M* | 792 | 52.02 | Zinc metalloprotease |
|  | *aph(6)-I* | 837 | 55.91 | APH(6)-I family aminoglycoside O-phosphotransferase |
|  | *aph(3'')-Ib* | 804 | 56.22 | Aminoglycoside O-phosphotransferase APH(3'')-Ib |
|  | *xerD* | 1014 | 61.14 | Tyrosine recombinase XerD |
|  | *endonuclease* | 642 | 49.07 | Restriction endonuclease |
